# Substance Use, General Health and Health Literacy as Predictors of Oral Health in Emerging Adult Sexual Minority Men of Color: A Secondary Data Analysis

**DOI:** 10.3390/ijerph18041987

**Published:** 2021-02-18

**Authors:** S. Raquel Ramos, David T. Lardier, Rueben C. Warren, Melba Cherian, Sarwat Siddiqui, Trace Kershaw

**Affiliations:** 1 Rory Meyers College of Nursing, New York University, New York, NY 10016, USA; mc4952@nyu.edu (M.C.); ss6367@nyu.edu (S.S.); 2 Department of Individual, Family, and Community Education, College of Education and Human Sciences, University of New Mexico, Albuquerque, NM 87131, USA; dlardier@unm.edu; 3 Department of Psychiatry and Behavioral Sciences, University of New Mexico School of Medicine, University of New Mexico, Albuquerque, NM 87131, USA; 4 National Center for Bioethics in Research and Health Care, Tuskegee University, Tuskegee, AL 36088, USA; rwarren@tuskegee.edu; 5 School of Public Health, Yale University, New Haven, CT 06520, USA; trace.kershaw@yale.edu; 6 Center for Interdisciplinary Research on AIDS, Yale University, New Haven, CT 06520, USA

**Keywords:** oral health, systemic health, tooth loss, health literacy, sexual minority men, substance use, electronic cigarette, young adult

## Abstract

There is limited evidence surrounding oral health in emerging adult, sexual minority men of color. This study examined the association between sociodemographic factors, health literacy, cigarette, e-cigarette, and alcohol use on oral health outcomes. Secondary data analysis was conducted with 322 sexual minority men ages 18–34 in the United States. Between-group, mean-level, and multivariable logistic regression analyses examined differences on oral health outcomes. Increased cigarette (aOR = 1.84, *p* = 0.03), e-cigarette (aOR = 1.40, *p* = 0.03), and alcohol use (aOR = 2.07, *p* = 0.05) were associated with extended time away from the dentist. Health literacy (aOR = 0.93, *p* = 0.05) was negatively associated. Increased cigarette (aOR = 1.17, *p* = 0.04) and cigarette use (aOR = 1.26, *p* = 0.04) were associated with tooth loss. Health literacy was negatively associated (aOR = 0.65, *p* = 0.03). Increased e-cigarette (aOR = 1.74, *p* = 0.04) and cigarette use (aOR = 4.37, *p* < 0.001) were associated with dental affordability issues. Lower health literacy and racial identification as Black were associated with dental affordability issues; demonstrating an urgent need to address these factors to improve oral health in emerging adult sexual minority men of color.

## 1. Introduction

Securing the health of gay and bisexual men (herein referred to as sexual minority men (SMM)), has historically been a challenge in the United States. The Centers for Disease Control and Prevention identified HIV/AIDS, sexually transmitted infections, and viral hepatitis as the main health issues concerning SMM [1]. These topics reflect the majority body of research impacting SMM, though there is a lack of research exploring other aspects of the health in this population. Multiple health disparities are well documented over the last two decades that revealed this population’s ongoing vulnerability to negative health outcomes, including those related to oral and systemic health [2]. Oral health is an area of concern in the general population, but there has been little attention reported on this topic among SMM. In this paper, we refer to oral health and dental health interchangeably.

Oral health is a largely understudied health topic in sexual minority communities. In 2000, the first ever-released statement from the Surgeon General on Oral Health in America reported the major associations between oral and systemic health [2,3,4]. Adverse oral health challenges include, but are not limited to dental caries, periodontal diseases, and oral cancers [4]. Poor oral health manifestations, such as gingivitis, bone loss around the teeth, oral candidiasis, and dental caries, may be of particular concern in the SMM community due to the prevalence of HIV [5]. Moreover, poor oral health has been linked to conditions such as endocarditis, cardiovascular disease, and pneumonia [6]. Research on the sexual minority communities has widely focused on general health disparities [7,8], and literature on oral health specific disparities among this population is limited; leaving sexual minority communities at a disadvantage because of the limited existing information published about their risk. With little evidence available on this subject, students and oral health professionals are not equipped to address the oral health risks and needs of this population.

While clinical measures were not statistically significant between sexual orientation, self-reported oral health measures indicated that sexual minority adults reported worse subjective oral health than their heterosexual counterparts [9]. In that study, 35% of sexual minority men reported more frequent concerns about “bone loss around teeth” when compared to bisexual (10%) and heterosexual (11%) men [9]. Additionally, 30% of bisexual participants reported a greater likelihood of facing barriers to access oral health care than heterosexual adults [9]. However, this study did not find a clinical basis for these variances in oral health focused assessments [9]. These findings from that study suggested that sexual minority groups are not homogenous, and that further research is needed to provide greater insights about the factors affecting issues on oral health, such as health literacy.

Health literacy is defined as the degree to which an individual can obtain, understand, and communicate information and services for basic health care needs [10]. Poor health literacy has been identified as a potential mediator of negative oral health outcomes. One study found that low oral health literacy was associated with dental caries and periodontal disease [11]. In addition, higher health literacy has been associated with the presence of more teeth and less plaque as compared to those with lower health literacy [12]. Low health literacy has also been found to be associated with failure to keep oral health appointments. One study found that low health literacy resulted in a two-fold increase in the risk of missed oral health appointments [13]. This implies that health literacy appears to play a significant role in oral health outcomes. It is also important to recognize that the associations between health literacy and clinical measures of oral health and frequency of oral health visits have not been specifically studied in SMM. We will address this gap in the literature, which will add to the limited data on improving oral health outcomes in this population.

Minority stress theory posits that sexual minority men experience a greater degree of social stress than heterosexual men [14]. This has resulted in the widespread prevalence of mental health disorders and behavioral health outcomes [15]. Stressful experiences include prejudice, stigma, negative self-schemas, expectation of rejection, and potential concealment of sexual identity. This manifests as emotional dysregulation, impaired coping processes, and a higher prevalence of mental health disorders and suicidality [15]. Sexual minority men of color experience discrimination, harassment, and violence at much higher rates, which is linked to smoking [16] and alcohol use [17]. When compounded, risk behaviors such as substance use can increase when sexual minority individuals also belong to a minoritized racial and/or ethnic group [18], making it critically important to examine tobacco and alcohol use as predictors of oral and general health in this population.

Tobacco use is a known factor contributing to oral disease [19] and remains a prevalent source of periodontal diseases, multiple oral cancers, and almost 9 out of 10 lung cancers [20]. Several studies have identified an increased prevalence of tobacco use in SMM compared to heterosexual men. In one study, 35.7% of gay men and 45.2% of bisexual men reported past-year cigarette smoking compared to 26.0% of heterosexual men [21]. The use of e-cigarettes is an increasingly common form of tobacco use. The 2012–2013 National Adult Tobacco Survey found that cigarette smoking and e-cigarette use were more prevalent in SMM [22]. Data collected from 8010 young adults outside of bars in 7 U.S cities found that there were higher rates of usage of alternative tobacco products (e-cigarettes, cigarillos, chewing tobacco, snus, and hookah), as well as dual use of cigarettes and other tobacco products, in SMM compared to heterosexual men [23]. Current use of tobacco has also been found to result in higher frequency of recent substance use and poorer mental, psychosocial, and general health [24].

Alcohol use is linked to an increased risk of oral cancer [25,26,27]. Alcohol use disorder (AUD) is especially prevalent amongst SMM, who are 1.5 times more likely to meet the criteria for alcohol dependence than their heterosexual counterparts [28]. Gay men aged 18–23 years old had the highest prevalence of AUD compared to all groups. Research in SMM with cancer and cancer survivors has displayed that greater alcohol use and binge drinking occur compared to their heterosexual peers [29]. Other studies have found higher periodontal diseases among men with harmful alcohol use and worse oral health for men and women with alcohol dependence [30]. The amount of alcohol consumption is inversely related to the number of outpatient medical visits [31]. In addition, heavy drinkers are more likely to be admitted to hospital or the emergency department, while using fewer preventative health services than light drinkers [32]. While existing data are about the utilization of general preventative health services, these findings may have associated implications for outpatient oral health care. People who engage in heavy alcohol consumption may potentially be at risk for underutilizing oral health care, which is of particular concern because of the prevalence of alcohol use in SMM [28]. To our knowledge, there is limited existing data on substance use and frequency of oral health visits among SMM of color. The purpose of this secondary data analysis was to examine the association between sociodemographic factors, health literacy, cigarette, e-cigarette, and alcohol use on oral health outcomes in a sample of sexual minority men of color ages 18–34.

## 2. Materials and Methods

In 2018, the primary study entitled, HIV Oral Testing Infographic Experiment, (HOTIE) was a mixed methods study with *n* = 322 SMM of color ages 18–34. In the primary study, an infographic for HIV self-testing was developed and tested. Details on The HOTIE Study are reported elsewhere^n.d^ (clinialtrials.gov NCT04061915). In this secondary data analysis, we were interested in how outcomes of oral health are impacted by behaviors and knowledge, such as substance use and health literacy. These predictors are important to assess when examining risk and knowledge in emerging adult SMM populations as they may uncover areas for future research on preventing poor oral health outcomes. The study was approved by the Institutional Review Board at New York University.

### 2.1. Measures

#### 2.1.1. Criterion Variables

General Health. General health was assessed using a single global health question derived from the Centers for Disease Control and Prevention Behavioral Risk Factor Surveillance System Survey Questionnaire [33]: “Would you say that in general your health is?” Responses were recorded on a five-point Likert scale from poor (1) to excellent (5), with a mean response of 3.82 (SD = 0.97), indicating “good” quality health.

Health Literacy. Health literacy was examined using the Short Assessment of Health Literacy-English, or SAHL-E. This is an 18-item measure designed to assess an English-speaker’s ability to read and understand common medical terms [34]. The test contains a printed common medical term, a key word (the correct response), and a distractor word. Responses were recorded dichotomously with either false (0) or true (1). Prior studies have demonstrated good internal consistency ranging from 0.80 to 0.89. For the current study, responses to 18-items were summed to yield a total score on health literacy ranging from 0 to 18 (M = 15.47, SD = 3.53). Higher scores indicated greater health literacy (Cronbach’s α = 0.89).

Cigarette, E-cigarette, and Alcohol Use. Cigarette, e-cigarette, and alcohol use question items were derived from the National Survey on Drug Use and Health [35]. Cigarette use was assessed using a single item to examine frequency of use: “Do you now smoke cigarettes every day, some days, or not at all?” Responses were collected using a three-point Likert scale from not at all (1) to every day (3), with a mean response of 2.70 (SD = 0.62). E-cigarette use was assessed using a single item to examine frequency of use: “Do you now use e-cigarettes or other electronic vaping products every day, some days, or not at all?” Responses were collected using a three-point Likert scale from not at all (1) to every day (3), with a mean response of 2.70 (SD = 0.55). Alcohol consumption was assessed using a single item to examine frequency of use during the past 30-days: “During the past 30 days, how many days per week or per month did you have at least one drink of any alcoholic beverage such as beer, wine, a malt beverage or liquor?” Responses were collected in a phrase-completion format, with mean response of 3.10 (SD = 3.29).

#### 2.1.2. Outcome Variables

Recent Dental Visit. Most recent dental visit was assessed using a single item derived from the Behavioral Risk Factor Surveillance System Survey Questionnaire [33] asking: “Including all types of dentists, such as orthodontists, oral surgeons, and all other dental specialists, as well as dental hygienists, how long has it been since you last visited a dentist or a dental clinic for any reason?” Responses were collected on a five-point scale: (1) Within the past year (anytime less than 12 months ago), (2) Within the past 2 years (1 year but less than 2 years ago), (3) Within the past 5 years (2 years but less than 5 years ago), (4) 5 or more years ago, and (5) Never. Participants mean responses were 1.96 (SD = 1.46). Ordinal responses were dichotomized with: 1 = last dental visit was 2 years or more/never, and 0 = within the past-year but less than 2-years. This dichotomized response was highly correlated with original ordinal response categories (r = 0.89, *p* < 0.001).

Dental Affordability and Access. Dental Affordability and Access was assessed using a single item from the Behavioral Risk Factor Surveillance System Survey Questionnaire [33] asking: “During the past 12 months, was there a time when you needed dental care but could not afford it?” Responses were collected dichotomously: Yes (1) and No (0), with a mean of 0.25 (SD = 0.43).

Tooth Loss. Tooth loss was assessed using a single item from the Behavioral Risk Factor Surveillance System Survey Questionnaire [33] asking: “Not including teeth lost for injury or orthodontics, how many of your permanent teeth have been removed because of tooth decay or gum disease?” Responses were collected on a four-point scale: No tooth loss (0) to All teeth (3). with a mean of 1.23 (SD = 0.53) and 16.8% of participants having lost at least one tooth. Ordinal responses were dichotomized with 0 = no tooth loss and 1 = tooth loss. This dichotomized response was highly correlated with original ordinal response categories (r = 0.91, *p* < 0.001).

#### 2.1.3. Covariates

Several sociodemographic covariates were tested as statistical controls. Covariates were included in fully specified multivariable models and retained based on performance in model. These covariates included age (in years), race-ethnicity, education completed, employment status, individual income, and current health insurance. Age was measured in years. Race-ethnicity was categorized dichotomously (Yes = 1, No = 0) using several questions that asked participants their race-ethnicity including Hispanic/Latinx, Black/African American, American Indian, Asian, and Middle Eastern. Participants had the option of choosing multiple racial-ethnic identities. Education completed was characterized using seven items, including less than high school (1), high school graduate/GED (2), some college (3), 2-year degree (4), 4-year degree (5), professional degree (e.g., Medical Doctor, Nurse; (6), and doctorate (e.g., Juris Doctorate, PhD). Most participants had either a high school diploma (23.9%), some college (23.3%), or 4-year degree or higher (40.1%). Individual income was categorized using 12-items ranging from less than $10,000 per year (1) to more than $150,000 per year (2). Approximately, 57% of participants had an income of $10,000 to $39,999, with 18% having an income of less than $10,000 per year and 3.4% having an income of more than $150,000 per year. Current health insurance was measured using six-items that included uninsured (1), private health insurance (2), state-sponsored health insurance (3), Medicaid (4), Military healthcare (e.g., Tricare, VA, CHAMP-VA; (5), and no health insurance (6). Over 50% of participants had either private insurance (36.3%) or no insurance (20.5%).

### 2.2. Statistical Analysis

Analyses were conducted in STATA v. 15.0 (StataCorp, College Station, TX, USA). Following descriptive analyses, ad hoc testing of demographic and other variables determined beneficial to identify any significant relationships that might warrant future study were examined. Between-group differences were examined using chi-square difference test to determine significant sociodemographic differences on outcomes of interest—recent dental visit, dental affordability, and tooth loss. Mean-level difference tests (*t*-test, ANOVA) were used to determine significant sociodemographic differences on domains of oral health—e-cigarette use, cigarette use, alcohol use, general health, and health literacy. In addition, mean-level between group difference tests were conducted on domains of oral health associated with recent dental visit, dental affordability, and tooth loss. Last, multivariable logistic regression analyses were used to examine results of the descriptive and comparative analyses. Selected variables with a *p* ≤ 0.20 were chosen to determine which demographic and domains of oral health were associated independently with recent dental visit and dental affordability. Traditional levels such as 0.05 can fail in identifying variables known to be important [36]. Variables were retained based on meaningful contribution and statistical significance to the final analytical model [37]. Adjusted odds ratios (aOR) are presented in the multivariable logistic regression model.

## 3. Results

### 3.1. Sociodemographic Characteristics and Outcomes and Domains of Oral Health

Table 1 highlights the sample sociodemographic characteristics. All participants were male, and self-identified as predominantly Hispanic (48.8%) and Black (37.9%). Participant ages ranged between 18 and 34 years (M = 26.35, SD = 4.66). Most participants were employed full-time (52.4%), and had either some college (23.3%), a 2-year college degree (11.8%), or a 4-year college degree or higher (40.1%). Most had an annual income (56.5%) that ranged from less than $10,000 per year to $39,999 per year. Approximately, 63.1% were either on their parent’s health insurance (16%), had private health insurance (36.4%), or state-sponsored insurance (10.7%); however, 20.7% had no health insurance.

On the outcome variable of most recent dental visit, there were several statistically significant differences (see Table 1). Statistically significant differences were noted on employment status (χ^2^ = 22.07, *p* < 0.001), education (χ^2^ = 20.57, *p* = 0.002), income (χ^2^ = 43.39, *p* < 0.001), and health insurance (χ^2^ = 27.05, *p* < 0.001). Similarly, on the outcome variable of dental affordability, statistically significant differences were noted among those participants that identified as Black (χ^2^ = 3.34, *p* = 0.05) and Asian (χ^2^ = 5.57, *p* = 0.02). No additional statistically significant differences were identified.

On identified domains of oral health, there were several statistically significant differences (see Table 2). For e-cigarette use, statistically significant mean-level differences were noted on Hispanic racial-ethnic identity (t (320) = −1.36, *p* = 0.04), employment status (F (4) = 4.34, *p* = 0.002), and health insurance (F (5) = 3.47, *p* = 0.005). Those self-employed (M_e-cigarette use_ = 1.58, SD = 1.58) and with military healthcare (M_e-cigarette use_ = 1.70, SD = 0.67) had slightly higher means on rates of e-cigarette use, when compared to those in other groups. For cigarette use, several statistically significant mean-level differences were identified on age (t (320) = −2.95, *p* < 0.001), employment (F (4) = 4.56, *p* = 0.001), and education (F (4) = 2.54, *p* = 0.02). Those who were 18 to 24 years of age (M_cigarette use_ = 0.37, SD = 0.69), unemployed (M_cigarette use_ = 0.50, SD = 0.82), and with less than a high school education (M_cigarette use_ = 0.80, SD = 1.09) had slightly higher means on rates of cigarette use, when compared to those in other groups. For alcohol use, statistically significant mean-level differences were found on age (t (320) = −2.56, *p* = 0.02), with those 25 to 34 years of age engaging in slightly higher 30-day alcohol use (Malcohol use = 0.80, SD = 1.09). In addition, regarding general health, statistically significant mean-level differences were noted on employment (F (4) = 5.22, *p* < 0.001) and health insurance (F (5) = 3.70, *p* = 0.003). Those who were unemployed (M_general health_ = 4.12, SD = 4.23) and had Medicaid (M_general health_ = 4.30, SD = 4.06) reported slightly higher general health.

### 3.2. Mean Level Group Differences on Substance Use and Health and Wellness Domains with Recent Dental Visit, Dental Affordability, and Tooth Loss

Participants self-reported various domains of oral health including cigarette use, e-cigarette use, and alcohol use, as well as general health and health literacy. Table 3 examines mean level differences of self-reported substances used and domains of health and wellness. Statistically significant results indicated (t (320) = 2.57, *p* = 0.01) that those who attended dental visits within the past year but less than 2-years had slightly higher cigarette use (M_cigarette use_ = 2.77, SD = 0.53), when compared to those who visited the dentist 2 years or more before being surveyed (M_cigarette use_ = 2.59, SD = 0.73). Statistically significant differences were also noted on variables of health and wellness, including general health (t (320) = −3.75, *p* < 0.001). Participants who attended dental visits within the past year but less than 2 year having slightly higher mean rates of general health (M_general health_ = 3.97, SD = 0.97). Statistically significant differences were also noted on health literacy (t (320) = 1.97, *p* = 0.05). Those who had extended time away from dentist—2 years or more before being surveyed had slightly higher mean responses of health literacy (M_health literacy_ = 15.84, SD = 3.31), when compared to those who attended dental visits within the past year but less than 2 years (M_health literacy_ = 15.04, SD = 3.89). No additional statistically significant differences were noted.

Table 4 examines mean level differences of self-reported substances used and domains of health and wellness on dental affordability and access. Statistically significant differences were identified on e-cigarette use (t (320) = 3.95, *p* < 0.001), with those who had dental affordability issues having slightly higher e-cigarette use (M_e-cigarette_ use = 1.50, SD = 0.65), when compared to those who did not have dental affordability issues (M_e-cigarette_ use = 1.33, SD = 0.49). Statistically significant differences were also noted on mean cigarette use (t (320) = −4.91, *p* < 0.001), with those who had no dental affordability issues reporting slightly higher cigarette use (M_cigarette use_ = 2.80, SD = 0.53). Statistically significant differences were further noted on variables of health and wellness including general health (t (320) = −3.36, *p* = 0.001), with participants who had no dental affordability issues reporting slightly higher means of general health (M_general health_ = 3.92, SD = 0.92), when compared to those with dental affordability issues (M_general health_ = 3.51, SD = 1.06). No additional statistically significant differences were noted.

Table 5 examines mean level differences of self-reported substances used and domains of health and wellness on tooth loss. Statistically significant results were present on e-cigarette use (t (320) = 0.86, *p* = 0.05), with those who had tooth loss having slightly higher e-cigarette use (M_e-cigarette use_ = 1.35, SD = 0.56), when compared to those who did not have tooth loss (M_e-cigarette use_ = 1.28, SD = 0.54). Statistically significant differences were also noted on mean cigarette use (t (320) = 0.83, *p* = 0.04), with those who had tooth loss reporting slightly higher cigarette use (M_cigarette use_ = 1.45, SD = 0.64). Statistically significant differences were also observed on variables of health and wellness, including health literacy (t (320) = 2.25, *p* = 0.03), with participants who had tooth loss reporting slightly lower means of health literacy (M_health literacy_ = 3.23, SD = 2.95), when compared to those with no tooth loss (M_health literacy_ = 3.64, SD= 3.50). No additional statistically significant differences were noted.

### 3.3. Exploratory Multivariable Logistic Regression Analyses: The Association between Substance Used, and Domains of Health and Wellness on Dental Health Outcomes

Exploratory multivariable logistic regression analyses (Table 6) examined the association between self-reported e-cigarette use, cigarette use and alcohol use, as well as domains of health and wellness on dental health outcomes. Age, race-ethnicity, employment status, education, income, and insurance type were included initially as covariates due to *p* ≤ 0.20 in between group analysis. Adjusted odds ratios (aOR) are reported. Cigarette use (aOR = 1.84, 95% confidence interval [CI] = 1.45, 3.66) and alcohol use (aOR = 2.07, 95% confidence interval [CI] = 1.12, 4.28) had a positive and significant association with most recent dental visit being 2-years or more before being surveyed. One way to interpret these findings is that for every one-unit increase in cigarette use, participants had 84% greater odds having attended the dentist 2-years or more before being surveyed. Similarly, participants who engaged in more alcohol use had 107% greater odds of attending their last dental visit 2 years ago or more before being surveyed. Those participants who used e-cigarettes were also likely to have extended time away from the dentist (aOR = 1.40, 95% CI = 1.04, 3.99). Furthermore, general health (aOR = 0.66, 95% CI = 0.41, 0.89) and health literacy (aOR = 0.93, 95% CI = 0.11, 0.98) were negatively associated with extended time away from the dentist. Several sociodemographic variables were also significant and negatively associated with time away from the dentist including income (aOR = 0.84, 95% CI = 0.55, 0.98), use of parent’s health insurance (aOR = 0.20, 95% CI = 0.11, 0.51), SCHIP insurance (aOR = 0.13, 95% CI = 0.09, 0.88), private insurance (aOR = 0.10, 95% CI = 0.03, 0.57), and using state sponsored insurance (aOR = 0.21, 95% CI = 0.05, 0.77). This model accounted for 30% of the variability in the outcome of recent dental visit.

Several notable outcomes were also identified on dental affordability. E-cigarette use (aOR = 1.74, 95% CI = 1.13, 3.83) and cigarette use (aOR = 4.37, 95% CI = 1.65, 10.85) had a positive and significant association with dental affordability issues. Interpretation of these findings indicates that for every one unit increase in e-cigarette use and cigarette use, participants had a 74% and 337% greater odds, respectively, of identifying need for dental health care but unable to afford treatment. Similarly, greater perceived general health (aOR = 1.68, 95% CI = 1.01, 4.15) and health literacy (aOR = 1.15, 95% CI = 1.11, 3.10) were associated with identifying need for dental health care but unable to afford treatment. Several sociodemographic variables were also significant and negatively associated with time away from the dentist. These included having less than a high school education (aOR = 0.13, 95% CI = 0.09, 0.88), a 2-year college degree (aOR = 0.11, 95% CI = 0.05, 0.52), and having a 4-year college degree (aOR = 0.07, 95% CI = 0.01, 0.88). Identifying as Black was positively associated with identifying need for dental health care but unable to afford treatment (aOR = 2.66, 95% CI = 1.61, 5.16). This model accounted for 26% of the variability in the outcome of dental affordability and access.

Last, and similar to previous findings on other outcomes, e-cigarette use (aOR = 1.17, 95% CI = 1.01, 3.33) and cigarette use (aOR = 1.26, 95% CI = 1.11, 2.73) were each positively associated with tooth loss. However, better overall general health was negatively associated with tooth loss (aOR = 0.65, 95% CI = 0.11, 0.86). In this model, identifying as Hispanic was negatively associated with tooth loss (aOR = 0.15, 95% CI = 0.01, 0.88). No additional sociodemographic variables were associated with the outcome of tooth loss. This model accounted for 22% of the variability in the outcome of tooth loss.

## 4. Discussion

The purpose of this study was to examine the predictors of oral health in a population of sexual minority men of color in the United States. Our findings provide insightful data on oral health in the SMM population, adding to what is known about the negative effect of tobacco use on tooth loss and the impact of social determinants of health, with positive oral health outcomes associated with health literacy and identifying as Black being a barrier to the access of oral health care. The influence of cigarette use, and e-cigarette use on oral health outcomes, was evident in this study. Both cigarette use and e-cigarette use were associated with tooth loss. This aligns with past findings that tobacco-use patterns were associated with worse periodontal health compared with tobacco never users [38]. A systematic review of the literature revealed that former smokers have a reduced risk of tooth loss when compared to current smokers [39]. Those who used cigarettes and e-cigarettes had greater odds of having attended the dentist 2-years or more before being surveyed. Likewise, those who engaged in more alcohol use were also likely to have extended time away from the dentist. Additionally, engaging in e-cigarette use and cigarette use increased the odds of identifying need for oral health care but the inability to afford treatment. It is important to note that we did not look at the use of other substances such as cannabis, cocaine, or polysubstance use, which have been linked to periodontitis [40]. Future research should examine the associations between these substances and oral health in SMM.

A unique contribution to the literature that our study findings demonstrated is the association of substance use to the frequency of dental visits and inability to afford treatment. This was a novel finding to which we have not identified other similar findings in literature on SMM of color. Past research has linked alcohol use to decreased outpatient visits including general preventative health care, but not to decreased oral health visits [31]. This is important because we have identified in our study that substance use is a factor that plays into underutilization of oral health care in SMM and must be addressed in efforts to improve oral health in this population. Further research is needed to elucidate the mechanisms behind these results as well as interventions to mitigate this risk factor.

E-cigarette use has recently increased among LGBTQ+ individuals, young adults, and those of low economic status [41]. The appeal of e-cigarettes to young adults is primarily attributed to trendy flavored tobacco, stylish device designs, social acceptability, and lower perceived health risks. However, adolescents who use e-cigarettes have an increased likelihood of progressing onto traditional cigarettes [41]. E-cigarettes are potentially just as harmful, as usage has been linked to acute lung injury [42]. Additionally, it was found that those who had dental affordability issues had slightly higher e-cigarette use compared to cigarette use. Part of the appeal of e-cigarettes may be that they are notably cheaper than traditional cigarettes. The low-cost of these products may make it a convenient option for individuals with lower incomes [43].

Health literacy was another factor that was examined in the context of oral health. Participants who had tooth loss reported lower means of health literacy. This finding is supported by existing literature that supports the association of lower health literacy to tooth loss [12]. In addition, lower health literacy scores were associated with time away from the dentist. This confirms existing findings that poor health literacy results in decreased utilization of oral health care [13]. With the results of this study, we were able to identify health literacy as a barrier to achieving positive oral health outcomes in sexual minority men of color, addressing the gap in current literature. Moving forward, further studies on improving oral health literacy in SMM of color is needed to improve oral health in this population.

Identifying as Black was positively associated with identifying the need for oral health care but having the inability to afford treatment. As such, those who identified as Black reported the highest proportion, 43.3%, of having seen the dentist 2 or more years before being surveyed and the lowest proportion, 56.6%, of having seen the dentist in the last 12 months, highlighting the pervasiveness of untreated oral health issues [44]. This aligns with national data from the Behavioral Risk Factor Surveillance System in 2018, which found that 69.7% of non-Hispanic whites accessed oral health care in the past year at a higher rate compared to 60.5% of those who identified as Black [33]. Our findings indicate that Blacks had the highest percentage of tooth loss, 28.7%, compared to other racial groups. Moreover, a report from 2004 indicated that Black men were 1.5 times more likely to have missing teeth when compared to their non-Hispanic white counterparts [44]. Data from the CDC indicated that Blacks, Hispanics, and American Indians and Alaska Natives have the poorest oral health of any racial and ethnic groups in the United States [45]. Likewise, a study in the US with a nationally representative sample found that Hispanic Whites experienced weaker oral health benefits from socioeconomic status as compared to non-Hispanic Whites [46]. Since the 2000 publication by the Surgeon General on Oral Health in America [3], not much has changed with regards to the higher prevalence of oral health disparities in those from of low socioeconomic status, low oral health literacy, and those from non-white ethnic and racial backgrounds [47]. Previous research in Iowa City confirmed that having a higher income, being White, and identifying as heterosexual were all associated with higher odds of having visited a dentist in the past year [48]. Through this study, we were able to ascertain that racial disparities in access to dental care and poor oral health do exist within a population of sexual minority men, highlighting the critical need to target interventions within this population.

### 4.1. Limitations

There were some limitations to this study. First, the data were obtained through a cross-sectional survey, which can only account for one point in time. As such, it would not reflect an individual’s constantly evolving health status and behaviors. Second, the survey elicited subjective, cross-sectional data, and we cannot make a direct conclusion about certain risk behaviors and its effect on health status. Third, we examined oral health through the lens of cigarette, e-cigarette, alcohol use, and health literacy, which are only a few of many influential factors that could affect oral health. Fourth, while we also obtained sociodemographic data, we were not able to look at other factors that may influence perception of oral health, such as comorbidities and psychosocial history. Fifth, as with all survey data, responses are subject to desirability bias and it is possible that some participants may have chosen to limit or enhance their responses. Sixth, the data analyzed were from an existing dataset. As such, our analysis was limited to the variables in the dataset and we did not examine the linkages between cocaine, cannabis, and polysubstance use. Seventh, the majority of our participants had an income of less than $40,000, which may have contributed to finding more negative oral health outcomes. Last, our small sample size limits generalizability of the findings to the broader population of those who identify as SMM of color. Nonetheless, the small sample size does not discount the findings nor the contribution this study provides to the literature about the association between sociodemographic factors and substance use on oral health outcomes in SMM of color.

### 4.2. Implications

Our results highlighted the underutilization of dental care and the negative oral health outcomes in SMM of color. In order to address this issue, we recommend enhancing the discussions between oral health providers and patients. Discussions with providers may possibly mitigate the risk of increased substance use that may be confounded by SMM’s lived experience with discrimination. Despite being a subgroup at greater risk, SMM are less likely to be offered advice about changing their drinking behavior in comparison to heterosexual men. In a sample of 65,265 individuals from the 2014 Behavioral Risk Factor Surveillance System survey [39], an estimated 25.5% of adults who attended a physical checkup were not questioned about their drinking habits [49]. Further, it was found that former smokers had a lower risk of tooth loss compared to current smokers [39], and that measures such as smoking cessation may also benefit oral health outcomes in this population.

Another important and relevant topic for oral health providers to discuss with SMM is prevention of oral human papilloma virus (HPV). In two studies with young adult SMM, over 8% of the sample’s participants tested positive for oral HPV [50,51], highlighting the relevance and importance of oral HPV prevention and screening efforts as an instrumental component of oral health. A study with a sample of community dwelling U.S adults ages 18–59, obtained from the National Health and Nutrition Examination Survey 2009–2014, reported a higher prevalence of oral HPV-positive status was found in gay and lesbian individuals (11.3%) in comparison to heterosexual (7.1%) and bisexual (8.6%) individuals [9]. As our data suggests, this is an oral health issue that impacts sexual minority populations and merits further attention as a point of discussion with patients.

Next, another important area to consider is health literacy as it is an important component of oral health management. Poor health outcomes in the US have been largely a result of low health literacy [52]. Our research findings support this by reporting the association between tooth loss and lower health literacy. This discrepancy may partly be due to the lack of formatting oral health-related information to the individualized learning needs of persons with low or varying levels of literacy [53]. Formal training in sexual health communication within an oral health lens, may promote enhanced patient understanding of the preventable oral risk factors that are creating disproportionate disparities.

Further, a curriculum for intensive oral health provider trainings on messaging and communication on decreasing substance use, prevention of oral HPV, and screening for HIV via oral HIV testing in a non-biased and affirming way is critically needed. This is vital to the success and sustainability of such an initiative. In a national survey of 1010 students from Schools of Medicine, Dental Medicine, and Nursing, dental students were less likely to report an interest in receiving formal LGBTQ+ health education [54]. Dental students displayed more stereotypical attitudes and less positive perceptions of formal training in LGBTQ+ health, suggesting a greater need for inclusion of the topic to address disparities in care. In a study on U.S. and Canadian dental schools, 76.6% of respondents reported a complete lack of education on LGBTQ+ health [55]. This gap in knowledge of providing culturally competent care proves to be a barrier when trying to foster trust between health care professionals and patients [55]. Intake forms with gender selection as male or female only and lack of LGBTQ+ friendly office design have proven to cause gender identity related stress and anxiety in transgender and gender nonconforming (TGNC) people. Modifications to treatment settings, such as including a nondiscrimination policy statement and LGTBQ flag can improve messaging and/or signage that is indicative of LGBTQ+ friendly, competent, and inclusive care [56]. Alliance building and partnering with LGBTQ+ organizations and community leaders is required as that starting point to successfully engage in this endeavor. This partnership is desperately needed to move the needle on these disparities and dismantle outdated traditions and long-held beliefs about oral health since not much has changed, as reported, over the last 20 years.

In sum, we recommend that oral health provider discussions with SMM of color place an emphasis on decreasing substance use, prevention of oral HPV, screening for HIV via oral HIV testing, and the updating antiquated academic curriculums to be LGBTQ+ inclusive. Our recommendations create many unique opportunities for oral health providers to promote oral and systemic health in this population. Building an infrastructure that supports integrating oral and systemic health as intersecting priorities to achieving overall health is greatly needed.

Lastly, COVID-19 is an ongoing topic of critical importance in oral health. Fear of exposure to COVID-19 at the early stages of the pandemic resulted in decreased patient volume at oral health care practices, as only 0.1% in a sample of 5000 were open with business as usual [57]. In a sample of 2195 dentists, fewer than 1% were estimated to be COVID-19 positive due to the implementation of enhanced infection prevention and control measures [57]. Despite this relative safety of oral health visits, patient volumes are now only at 65% of pre-COVID levels as of June 2020 [58]. This may be due to lack of affordability and accessibility to oral health care, which is still not considered an essential health benefit under the Affordable Care Act (ACA). This is especially concerning, as sexual minorities are at higher odds of delaying or not receiving care due to cost. COVID-19 has compounded the effects of delayed oral health care and may contribute to perpetuating poor oral health outcomes in this population.

## 5. Conclusions

Cigarette and e-cigarette use were evident as factors associated with tooth loss and merit further actions to mitigate, with special attention on methods of mitigation for a population of sexual minority men. Further research is needed to elucidate what factors motivate individuals to visit the dentist, in addition to a general need for oral health research on SMM. There is also a need for more inclusive care and provider discussions about health promotion behavior in relation to alcohol and tobacco use, considering the health literacy of all patients. Inequity in health care remains a prevalent obstacle in achieving better health outcomes, particularly among Black SMM. Furthermore, the long-term implications of the COVID-19 pandemic on the decreased utilization of oral health care is concerning and should be subject to further research and interventions.

## Figures and Tables

**Table 1:** Sociodemographic characteristics on oral health outcomes (*n* = 322).

		Most Recent Dental Visit				Dental Affordability Access				Tooth Loss			
	Total	Less Than 12 Months	More Than 2 Years Ago	Test Statistic (χ^2^)	*p*-Value	Yes, Affordability Issues	No, Affordability Issues	Test Statistic (χ^2^)	*p*-Value	No Teeth Loss	Teeth Loss	Test Statistic (χ^2^)	*p*-Value
**Gender**													
Male	322 (100%)	192 (59.6%)	130 (40.40%)	---	---	82 (25.5%)	240 (74.5%)	---	---	252 (78.3%)	70 (21.7%)	---	---
**Age** (M = 26.35, SD = 4.66)				0.91	0.23			0.63	0.23			0.65	0.41
18 to 24 years	114 (35.4%)	72 (63.2%)	42 (36.8%)			32 (28.1%)	82 (71.9%)			92 (80.7%)	22 (19.3%)		
25 to 34 years	208 (64.6%)	120 (57.7%)	88 (42.3%)			50 (24.0%)	158 (76.0%)			159 (76.8%)	48 (23.2%)		
**Race-Ethnicity ^a^**													
Hispanic	157 (48.8%)	98 (62.4%)	59 (37.6%)	0.99	0.22	38 (24.2%)	119 (75.8%)	0.25	0.22	123 (78.3%)	34 (21.7%)	0.01	0.86
Black	122 (37.9%)	69 (56.6%)	53 (43.3%)	0.77	0.20	38 (31.1%)	84 (68.9%)	3.34	**0.05**	87 (71.3%)	35 (28.7%)	5.57	**0.01**
White	74 (23.0%)	47 (63.5%)	27 (36.5%)	0.60	0.24	16 (21.6%)	58 (78.4%)	0.75	0.21	59 (79.7%)	15 (20.3%)	0.22	0.72
Asian Identity	69 (21.4%)	44 (63.8%)	25 (36.2%)	0.63	0.43	10 (14.5%)	59 (85.5%)	5.57	**0.02**	61 (88.4%)	8 (11.6%)	5.31	**0.02**
American Indian/Native American Identity	17 (5.3%)	11 (64.7%)	6 (35.3%)	0.19	0.66	5 (29.4%)	12 (70.6%)	0.14	0.70	13 (76.5%)	4 (23.5%)	0.03	0.85
Middle Eastern Identity	10 (3.1%)	9 (90.0%)	1 (10.0%)	1.95	0.14	3 (30.0%)	7 (70.0%)	0.11	0.74	8 (80.0%)	2 (20.0%)	0.02	0.89
**Employment**				22.07	**<0.001**			1.33	0.85			7.34	0.13
Employed Full-time	167 (51.8%)	119 (71.3%)	48 (28.7%)			40 (240%)	127 (76.0%)			132 (79%)	35 (21.0%)		
Employed Part-time	54 (16.8%)	28 (51.9%)	26 (48.1%)			16 (29.6%)	38 (70.4%)			37 (68.5%)	17 (31.5%)		
Self-employed	19 (6.0%)	7 (36.8%)	12 (63.2%)			6 (31.6%)	13 (68.4%)			13 (68.4%)	6 (31.6%)		
Unemployed	34 (10.5%)	13 (38.2%)	21 (61.8%)			9 (26.5%)	25 (73.5%)			29 (85.3%)	5 (14.7%)		
Student	45 (13.9%)	24 (53.3%)	21 (46.7%)			10 (22.2%)	35 (77.8%)			39 (86.7%)	6 (13.3%)		
**Education**				20.57	**0.002**			6.81	0.33			7.14	0.28
Less than high school	5 (1.6%)	2 (40.0%)	3 (60.0%)			2 (40.0%)	3 (60.0%)			5 (100%)	0 (0.0%)		
High school graduate/GED	77 (23.9%)	38 (49.4%)	39 (50.6%)			18 (23.4%)	59 (76.6%)			59 (76.6%)	18 (23.4%)		
Some College	75 (23.3%)	35 (49.3%)	38 (50.7%)			23 (30.7%)	52 (69.3%)			53 (70.7%)	22 (29.3%)		
2-year degree	38 (11.8%)	20 (52.6%)	18 (47.4%)			13 (34.2%)	25 (65.8%)			33 (86.8%)	5 (13.2%)		
4-year degree or higher	129 (40.1%)	95 (73.6%)	34 (26.4%)			26 (20.2%)	103 (79.8%)			102 (80.31%)	25 (19.69)		
**Income**				36.75	**<0.001**			9.92	0.43			4.01	0.67
Less than $10,000	59 (18.3%)	29 (49.2%)	30 (50.8%)			17 (d28.8%)	42 (71.2%)			46 (78%)	13 (22.0%)		
$10,000–$29,999	84 (26.1%)	33 (39.3%)	51 (23.1%)			27 (32.1%)	57 (67.9%)			62 (73.8%)	22 (26.2%)		
$30,000–$49,999	69 (21.4%)	43 (62.3%)	26 (37.7%)			20 (29.0%)	49 (71.0%)			54 (78.3%)	15 (21.7%)		
$50,000–$69,999	44 (13.7%)	34 (77.3%)	10 (22.7%)			8 (18.2%)	36 (81.8%)			33 (75%)	11 (25.0%)		
$70,000–$89,999	29 (9.0%)	23 (79.3%)	6 (20.7%)			4 (13.8%)	25 (86.2%)			25 (86.2%)	4 (13.8%)		
$90,000–$149,000	26 (8.1%)	23 (88.5%)	3 (11.5%)			6 (23.1%)	20 (76.9%)			23 (88.5%)	3 (11.5%)		
More than $150,000	11 (3.4%)	7 (63.6%)	4 (36.4%)			0 (0.0%)	11 (4.6%)			9 (81.8%)	1 (18.2%)		
**Insurance**				27.05	**<0.001**			7.21	0.21			2.05	0.84
Parent’s Health Insurance	51 (15.8%)	30 (48.8%)	21 (41.2%)			11 (21.6%)	40 (78.4%)			40 (78.4%)	11 (21.6%)		
Private Health Insurance	117 (36.3%)	86 (73.5%)	31 (26.5%)			27 (23.1%)	90 (76.9%)			90 (76.9%)	27 (23.1%)		
State Sponsored Health Plan	35 (10.9%)	23 (65.7%)	12 (34.3%)			13 (37.1%)	22 (62.9%)			22 (62.9%)	13 (37.1%)		
Medicaid	43 (13.4%)	24 (55.8%)	19 (44.2%)			25	53			31 (72.1%)	12 (27.9%)		
Military Health Care (TRICARE/VA/CHAMP—VA)	10 (3.1%)	6 (60.0%)	4 (40.0%)			5 (50.0%)	5 (50.0%)			5 (50%)	5 (50%)		
No Health Insurance	66 (20.5%)	23 (34.8%)	43 (65.2%)			14 (21.2%)	52 (78.8%)			52 (78.8%)	14 (21.2%)		

*Note*. ^a^ Participants had the option of choosing multiple options on race-ethnicity, with response options either no (0) or yes (1) for each race-ethnicity. Bold *p*-values indicate significance.

**Table 2:** Sociodemographic characteristics on domains of oral health (*n* = 322).

	E-Cigarette Use ^c^			Cigarette Use ^c^			Alcohol Use ^c^			General Health ^c^			Health Literacy ^c^		
	M (SD)	Difference Test	*p*-Value	M (SD)	Difference Test	*p*-Value	M (SD)	Difference Test	*p*-Value	M (SD)	Difference Test	*p*-Value	M (SD)	Difference Test	*p*-Value
**Gender**															
Male	1.30 (0.55)			0.29 (0.62)			3.10 (3.29)			3.82 (0.96)			3.55 (3.39)		
**Age** (M = 26.35, SD = 4.66)		−0.35	0.72 ^a^		−2.95	**<0.001 ^a^**		−2.26	**0.02 ^a^**		0.16	0.86 ^a^		−0.85	0.39 ^a^
18 to 24 years	1.28 (0.54)			0.37 (0.69)			2.17 (1.98)			3.82 (0.98)			3.31 (3.16)		
25 to 34 years	1.31 (0.55)			0.16 (0.41)			3.46 (3.64)			3.81 (0.97)			3.64 (3.46)		
**Race-Ethnicity ^d^**															
Hispanic	1.34 (0.56)	−1.36	**0.04 ^a^**	0.29 (0.610	0.07	0.93 ^a^	3.44 (4.03)	−1.34	0.18 ^a^	3.84 (1.00)	−0.42 ^a^	0.67 ^a^	3.43 (3.14)	0.61	0.54 ^a^
Black	1.35 (0.50)	−1.27	0.20 ^a^	0.35 (0.67)	−1.35	0.17 ^a^	2.67 (2.24)	1.30	0.19 ^a^	3.92 (0.91)	−1.46	0.14 ^a^	3.75 (3.63)	−0.79	0.43 ^a^
White	1.23 (0.48)	1.19	0.23 ^a^	0.27 (0.61)	0.37	0.71 ^a^	3.34 (3.61)	−0.55	0.58 ^a^	3.78 (1.01)	0.35	0.73 ^a^	3.18 (2.71)	1.09	0.27 ^a^
Asian Identity	1.19 (0.43)	1.85	0.06 ^a^	0.17 (0.48)	1.87	0.06 ^a^	3.28 (3.29)	−0.31	0.76 ^a^	3.72 (1.01)	0.88	0.38 ^a^	3.17 (3.21)	1.05	0.27 ^a^
American Indian/Native American Identity	1.35 (0.49)	−0.43	0.66 ^a^	0.29 (0.62)	0.02	0.98 ^a^	4.09 (5.59)	−1.02	0.31 ^a^	3.82 (0.97)	0.47	0.63 ^a^	3.29 (3.41)	0.32	0.74 ^a^
Middle Eastern Identity	1.70 (0.66)	−2.36	0.02 ^a^	0.500 (0.52)	−1.04	0.29 ^a^	2.50 (1.76)	0.45	0.65	3.80 (.97)	−1.93	0.05 ^a^	2.80 (1.39)	0.71	0.47 ^a^
**Employment**		4.34	**0.002 ^b^**		4.56	**0.001 ^b^**		1.50	0.21 ^b^		5.22	**<0.001 ^b^**		0.34	0.85 ^b^
Employed Full-time	1.35 (0.60)			0.34 (0.64)			3.25 (3.69)			3.51 (3.23)			4.01 (0.92)		
Employed Part-time	1.24 (0.47)			0.16 (0.46)			3.72 (3.02)			3.65 (3.66)			3.56 (1.00)		
Self-employed	1.58 (0.60)			0.47 (0.77)			3.43 (3.34)			3.53 (4.24)			3.58 (1.02)		
Unemployed	1.18 (0.45)			0.50 (0.82)			1.71 (1.64)			4.12 (4.23)			3.33 (0.99)		
Student	1.07 (0.25)			0.02 (0.14)			1.77 (1.36)			3.24 (2.68)			3.82 (0.94)		
**Education**		0.65	0.68 ^b^		2.54	**0.02 ^b^**		1.68	1.29 ^b^		0.96	0.44 ^b^		0.30	0.94 ^b^
Less than high school	1.40 (0.89)			0.80 (1.09)			2.67 (1.52)			4.20 (0.83)			3.60 (2.30)		
High school graduate/GED	1.25 (0.49)			0.31 (0.63)			1.75 (1.79)			3.88 (0.97)			3.96 (3.96)		
Some College	1.32 (0.62)			0.40 (0.71)			3.39 (3.65)			3.70 (0.93)			3.31 (3.31)		
2- year degree	1.32 (0.58)			0.39 (0.71)			2.33 (2.02)			3.63 (1.03)			3.55 (3.47)		
4-year degree or higher	1.35 (0.56)			0.23 (0.52)			3.95 (3.99)			3.87 (0.94)			3.49 (3.11)		
**Income**		0.57	0.87 ^b^		0.88	0.56 ^b^		1.70	0.07 ^b^		1.56	0.10 ^b^		0.63	0.81 ^b^
Less than $10,000	1.19 (0.48)			0.22 (0.59)			1.73 (1.20)			3.74 (1.02)			3.09 (2.61)		
$10,000–$29,999	1.21 (0.47)			0.34 (0.73)			2.42 (0.29)			3.57 (0.91)			3.97 (3.95)		
$30,000–$49,999	1.39 (0.68)			0.37 (0.63)			3.46 (4.38)			3.71 (1.25)			3.32 (2.98)		
$50,000–$69,999	1.33 (0.56)			0.08 (0.28)			1.89 (1.56)			4.00 (0.83)			2.71 (2.47)		
$70,000–$89,999	1.42 (0.61)			0.36 (0.68)			4.50 (4.89)			3.84 (0.89)			3.32 (1.66)		
$90,000–$149,000	1.36 (0.63)			0.28 (0.47)			2.50 (2.99)			4.36 (0.63)			4.36 (4.73)		
More than $150,000	1.18 (0.40)			---			3.75 (2.06)			4.45 (0.68)			4.27 (3.13)		
**Health Insurance**		3.47	**0.005 ^b^**		1.91	0.09 ^b^		0.76	0.57 ^b^		3.70	**0.003 ^b^**		0.94	0.46 ^b^
Parent’s Health Insurance	1.20 (0.44)			0.06 (0.24)			2.00 (1.44)			3.78 (0.86)			3.02 (2.35)		
Private health Insurance	1.38 (0.62)			0.37 (0.66)			3.28 (2.36)			3.91 (1.05)			3.60 (3.73)		
State Sponsored Health Plan	1.31 (0.53)			0.34 (0.68)			2.94 (2.56)			3.34 (0.93)			2.94 (2.07)		
Medicaid	1.33 (0.57)			0.34 (0.68)			3.11 (2.86)			4.16 (0.89)			4.30 (4.06)		
Military Health Care (TRICARE/VA/CHAMP—VA)	1.70 (0.67)			0.20 (0.63)			3.00 (0.89)			4.10 (0.74)			4.00 (3.68)		
No Health Insurance	1.12 (0.42)			0.30 (0.63)			3.65 (4.75)			3.65 (0.99)			3.65 (3.48)		

*Note*. M = Mean. SD = Standard deviation. ^a^ independent sample *t*-test conducted; ^b^ ANOVA conducted; ^c^ Raw scores used for analyses; ^d^ Participants had the option of choosing multiple options on race-ethnicity, with response options either no (0) or yes (1) for each race-ethnicity. Bold *p*-values indicate significance.

**Table 3:** Mean-level group differences between substance use and health and wellness domains, and the outcome of most recent dental visit (*n* = 322).

	Most Recent Dental Visit				
	Within the Past-Year But Less Than 2-Years M (SD)	More Than 2 Years AgoM (SD)	*t* (320 ^b^)	*p*-Value	Mean Difference (95% CI)
**Substance Use ^a^**					
E-cigarette Use	1.31 (0.56)	1.28 (0.53)	0.47	0.63	0.03 (-0.09, 115)
Cigarette Use	2.77 (0.53)	2.59 (0.73)	2.57	**0.01**	0.18 (0.04, 0.32)
Alcohol Use	1.83 (0.95)	1.94 (0.95)	−0.09	0.91	−0.01 (0.11, −0.22)
**Health and Wellness ^a^**					
General Health	3.97 (0.97)	3.59 (0.94)	−3.75	**<0.001**	−0.39 (−0.59, −0.18)
Health literacy	15.04 (3.89)	15.84 (3.31)	1.97	**0.05**	−0.79 (−1.61, −0.03)

*Note*. M = Mean. SD = Standard deviation. CI = Confidence interval. ^a^ Raw scores of variables used for analyses; ^b^ Degree of freedom. Bold *p*-values indicate significance.

**Table 4:** Mean-level group differences between substance use domains and health and wellness domains, and the outcome of dental affordability (*n* = 322).

	Dental Affordability Access				
	Yes, Affordability Issues	No, Affordability Issues	t (320 ^b^)	*p*–Value	Mean Difference
	*M (SD)*	*M (SD)*			(95% CI)
**Substance Use ^a^**					
E-cigarette Use	1.50 (0.65)	1.33 (0.49)	3.95	**<0.001**	0.27 (0.13, 0.40)
Cigarette Use	2.42 (0.77)	2.80 (0.53)	−4.91	**<0.001**	−0.37 (−0.53, −0.23)
Alcohol Use	1.79 (0.92)	1.86 (0.94)	−0.56	0.2	−0.06 (−0.31, 0.17)
**Health and Wellness ^a^**					
General Health	3.51 (1.06)	3.92 (0.92)	−3.36	**0.001**	−0.41 (−0.65, −0.17)
Health literacy	15.68 (3.18)	15.25 (3.84)	0.91	0.36	0.42 (−0.42, 1.27)

*Note*. M = Mean. SD = Standard deviation. CI = Confidence interval. Bold *p*-values indicate significance. ^a^ Raw scores of variables used for analyses; ^b^ Degree of freedom.

**Table 5:** Mean-level group differences between substance use domains and health and wellness domains, and the outcome of teeth loss (*n* = 322).

	Tooth Loss				
	No Tooth Loss M (SD)	Tooth Loss M (SD)	t (320 ^b^)	*p*-Value	Mean Difference (95% CI)
**Substance Use ^a^**					
E-cigarette Use	1.28 (0.54)	1.35 (0.56)	0.86	**0.05**	0.06 (0.001, 0.10)
Cigarette Use	1.27 (0.61)	1.45 (0.64)	0.83	**0.04**	0.06 (0.03, 0.13)
Alcohol Use	3.09 (3.44)	3.15 (2.69)	−0.09	0.92	−0.06 (−1.33, 1.21)
**Health and Wellness ^a^**					
General Health	3.88 (0.91)	3.59 (1.13)	0.90	0.36	0.41 (−0.48, 1.31)
Health literacy	3.64 (3.50)	3.23 (2.95)	2.25	**0.03**	0.13 (0.04, 0.55)

*Note*. M = Mean. SD = Standard deviation. CI = Confidence interval. ^a^ Raw scores of variables used for analyses; ^b^ Degree of freedom. Bold *p*-values indicate significance.

**Table 6:** Multivariable logistic regression analyses between substance use domains and health and wellness domains and dental health outcomes (*n* = 322).

	Most Recent Dental Visit				Dental Affordability Access				Tooth Loss			
	aOR	95% CI		Sig.	aOR	95% CI		Sig.	aOR	95% CI		Sig.
		LL	UL			LL	UL			LL	UL	
**Substance Use**												
E-cigarette Use	1.40	1.04	3.99	**0.03**	1.74	1.13	3.83	**0.04**	1.17	1.01	3.33	**0.04**
Cigarette Use	1.84	1.45	3.66	**0.03**	4.37	1.65	10.85	**<0.000**	1.26	1.11	2.73	**0.04**
Alcohol Use	2.07	1.12	4.28	**0.05**	1.04	0.41	2.82	0.89	0.98	0.45	2.97	0.83
**Health and Wellness**												
General Health	0.66	0.41	0.89	**0.001**	1.68	1.01	4.15	**0.003**	0.65	0.11	0.86	**0.03**
Health literacy	0.93	0.11	0.98	**0.05**	1.15	1.11	3.10	**0.05**	1.03	0.92	1.19	0.57
**Age**	1.06	0.95	1.14	0.26	0.66	0.90	1.10	0.46	1.11	0.79	1.19	1.08
**Race-Ethnicity**												
Hispanic	0.89	0.70	6.14	0.26	0.77	0.37	3.89	0.54	0.15	0.01	0.88	**0.05**
Black	1.88	0.11	7.81	0.20	2.66	1.61	5.16	0.01	2.32	1.09	9.51	0.11
White	1.75	0.38	6.80	0.41	1.21	0.19	3.74	0.71	2.13	0.22	4.44	0.22
**Employment**												
Full-time	0.26	0.24	4.93	0.12	1.90	0.25	5.49	0.27	3.23	0.31	6.77	0.34
Part-time	0.60	0.04	1.26	0.54	1.39	0.07	3.50	0.58	4.53	0.59	15.83	0.22
Self-employed	0.43	0.35	224.30	0.62	3.34	0.03	6.68	0.18	3.92	0.40	123.75	0.31
Unemployed	0.25	0.01	0.73	0.15	1.32	0.09	4.76	0.70	0.66	0.21	8.69	0.33
**Education**												
Less than high school	0.73	0.20	8.88	0.77	0.13	0.09	0.88	**0.05**	1.33	0.40	88.74	0.80
High school graduate/GED	0.71	0.27	11.80	0.74	0.17	0.13	6.48	0.10	1.55	0.11	17.72	0.83
Some college	0.64	0.47	15.96	0.57	0.24	0.88	0.11	0.20	1.66	0.39	41.54	0.77
2-year college	0.23	0.34	14.88	0.17	0.11	0.05	0.52	**0.04**	1.50	0.10	23.08	0.79
4-year college or more	0.26	0.46	11.08	0.24	0.07	0.01	0.88	**0.02**	1.05	0.47	45.19	0.81
**Income**	0.84	0.55	0.98	**0.05**	1.01	0.71	2.02	0.80	0.93	0.71	4.02	0.47
**Insurance**												
Under parents’ health insurance	0.20	0.11	0.51	**0.05**	0.81	0.83	20.40	0.74	1.40	0.31	5.94	0.71
SCHIP (CHIP/Children’s Health Insurance Plan)	0.13	0.09	0.88	**0.01**	1.24	0.11	6.6	0.69	1.44	0.33	8.15	0.70
Private Insurance	0.10	0.03	0.57	**0.01**	0.56	0.25	2.96	0.35	1.22	0.21	2.53	0.85
State-sponsored insurance	0.21	0.05	0.77	**0.03**	0.91	0.06	3.09	0.89	2.23	0.27	8.86	0.52
Nagelkerke R^2^	0.30				0.26				0.22			
Cox & Snell R^2^	0.25				0.20				0.18			

*Note*. aOR = adjusted odds ratio. CI = Confidence interval. Sig. = Significance. Bold *p*-values indicate significance.

## Data Availability

The data presented in this study are not publicly available due to privacy and ethical considerations on protecting sexual identity and HIV status of the study participants.
